# Factors related to autonomy among Lebanese women: a web-based cross-sectional study

**DOI:** 10.1186/s12905-021-01501-3

**Published:** 2021-10-20

**Authors:** Sandrella Bou Malhab, Hala Sacre, Diana Malaeb, Nathalie Lahoud, Dalia Khachman, Joelle Azzi, Chadia Haddad, Pascale Salameh

**Affiliations:** 1 INSPECT-LB (Institut National de Santé Publique, d’Épidémiologie Clinique et de Toxicologie-Liban), Beirut, Lebanon; 2 School of Pharmacy, Lebanese International University, Beirut, Lebanon; 3 Faculty of Public Health, Lebanese University, Fanar, Lebanon; 4 Faculty of Pharmacy, Lebanese University, Beirut, Lebanon; 5 Clinical and Epidemiological Research Laboratory, Faculty of Pharmacy, Lebanese University, Hadat, Lebanon; 6 Research Department, Psychiatric Hospital of the Cross, P.O. Box 60096, JalEddib, Lebanon; 7 INSERM, Univ. Limoges, CH Esquirol, IRD, U1094 Tropical Neuroepidemiology, Institute of Epidemiology and Tropical Neurology, GEIST, Limoges, France; 8 University of Nicosia Medical School, Nicosia, Cyprus; 9 Faculté de santé, Université Sainte Famille, Batroun, Lebanon

**Keywords:** Women, Independence, Autonomy, Anxiety, Stress, Depression

## Abstract

**Background:**

Autonomy involves making independent decisions and creating lasting and equitable power relationships within families. Many factors, dependent on both the woman and her partner, can influence self-dependence, and subsequent decision-making, exerting a protective or triggering effect on its development. Therefore, the primary objective of the study was to assess autonomy in a sample of Lebanese women. The secondary objective was to evaluate the association between socioeconomic status, psychological factors, and autonomy.

**Methods:**

A web based cross-sectional online study was conducted between June 8 and August 1, 2020. The questionnaire developed on Google Forms was distributed through social media and WhatsApp groups, using the snowball technique. The Women’s Autonomy Index (WAI) was created using three items adapted from a previous study. In addition, the Composite Abuse Scale Revised—Short Form (CASR-SF) was used to assess three domains of abuse: physical, sexual, and psychological. The Perceived stress scale short version to measure stress perception, the Lebanese Anxiety Scale to measure anxiety and the Patient Health Questionnaire (PHQ-9) to assess depression. The Statistical Package for the Social Sciences (SPSS) software version 25 was used for data analysis. Linear regressions were performed, taking the Women’s Autonomy Index as the dependent variable.

**Results:**

The sample consisted of 369 Lebanese women**.** University education level (beta = 1.263), alcohol consumption (beta = 0.586), intermediate income level (beta = 0.702), high income (beta = 0.911), employment (beta = 0.559), and older age (beta = 0.033) were significantly associated with higher WAI. Living in South Lebanon (beta = − 0.668) and being Druze (beta = − 323) were associated with lower WAI. Significantly higher mean scores of anxiety and perceived stress were found among women with low autonomy.

**Conclusion:**

In Lebanon, the autonomy of women depends on several personal and partner-related characteristics (education, socioeconomic status, age), in addition to the cultural (geographic and religious) environment. Furthermore, low autonomy is associated with higher perceived stress and anxiety and probable depression and domestic abuse.

**Supplementary Information:**

The online version contains supplementary material available at 10.1186/s12905-021-01501-3.

## Introduction

Autonomy or self-dependence is the ability to make decisions for oneself, to control one's own body, and to determine how to use resources without needing to consult or seek permission from another person [1]. In women, it can be viewed as the control over their own lives, materials, access to knowledge and information, and having equal contribution and opinions with their husbands or partners on matters affecting them and their families [1–3]. Autonomy involves making independent decisions, overcoming the constraints of physical mobility, and creating lasting and equitable power relationships within families [4].

The few studies that assessed this topic in developing countries suggest a close link between autonomy and the sociodemographic characteristics of women and the social environment in which they live. Many factors, dependent on both the woman and her partner, can influence self-dependence, and subsequent decision-making, exerting a protective or triggering effect on its development [5]. Indeed, education [6], employment and high household income [1, 7], older age [8], residing in urban areas [9], living with an educated and employed partner [1, 10], and having good marital relationships [11] are all predictors of greater autonomy in women, increasing their self-confidence, assertiveness, and decision-making. Oppositely, poverty [2] and religion [8] exacerbate their dependence and decrease their self-esteem. Furthermore, a few studies showed that women’s autonomy, including decision-making, is associated with psychological factors such as anxiety, stress, and depression [12–14].

In Lebanon, despite many vested benefits and rights, Lebanese women face discrimination on many levels, rooted in law and regulations, sectarian considerations, religious socio-cultural values differing according to religions, decision-making structures, public policies, development strategies, ongoing conflicts, security issues, and a rise in social conservatism. The absence of women from decision-making is mainly due to the patriarchal character of the society governed by customary and religious beliefs rather than codified law [15]. Various sects and religious communities, traditionally dominated by male members of the leading families, undermines the possibility of involvement of women in the decision process [15].

All these factors enhance the vulnerability of women and make gender equality in Lebanon an elusive goal. Furthermore, the economic system in Lebanon relies on minimal taxation and a commitment to the free market, favoring the private sector by giving it strong dominance over vital public services, including education, thus resulting in a significant gap in educational outcomes between public and private education, which reduces equal opportunities for children [15].

Our research hypothesis was that higher education, higher monthly income, Christian religion, being employed, and low violence are associated with higher autonomy, which, in turn, is associated with lower stress, anxiety, and depression. Therefore, the primary objective of the study was to assess autonomy in a sample of Lebanese women. The secondary objective was to evaluate the association between socioeconomic status, psychological factors, and autonomy.

## Methods

### Study design and sampling

A web based cross-sectional study conducted online between June 8 and August 1, 2020, using the snowball technique from all Lebanese regions (Beirut, Mount Lebanon, North, South, and Beqaa). Inclusion criteria were married women, aged 18 to 51, living with their partner, and with internet access. Exclusion criteria were single, widowed, or divorced women and those with fertility problems. The eligibility criteria were available in the consent section at the beginning of the online survey that required 40 min to complete. The questionnaire was pilot-tested on ten women to check the clarity, and the data included in the final database. Participation was anonymous and voluntary, and participants received no compensation for joining the study.

### Procedure

The online survey consisted of closed-ended questions in English and Arabic. The questionnaire was developed on Google Forms, and the link was distributed through social media and WhatsApp groups.

Approval was obtained from the ethics committee of The Psychiatric Hospital of the Cross (HPC-018-2020). All procedures were performed in accordance with relevant guidelines. Also, the procedure of this article followed the Strobe statement checklist (Additional file 1). 

### Sample size calculation

The sample size was calculated through two methods using the G-power software.

In the first method, the calculation considered the effect size between two means (abused and non-abused women) of the Women’s Autonomy Index. Based on an effect size of 0.56, an alpha error of 5%, and a power of 96%, the minimum sample required was 140 participants.

In the second method, the calculation was based on the number of predictors to be entered in the multivariable analysis. The minimum sample required was 311 women, based on an effect size f2 = 2%, an alpha error of 5%, a power of 80%, and taking into consideration 17 factors to be entered in the multivariable analysis.

The authors adopted the second calculation method since it yielded a higher sample size allowing for better analysis. The final sample size comprised of 369.

The variables used to perform the calculation are described in the questionnaire section below.

### Questionnaire

The questionnaire included two sections (Additional file 2). The first assessed the sociodemographic features of women and those of their partners as reported by the respondent, such as age, educational level, the region of residence, the number of rooms and the number of people living in the house, religion, working status, monthly income, smoking, alcohol status, duration of confinement, and physical activity. The confinement period corresponds to the sanitary lockdown mandated by the Lebanese government to face the COVID-19 pandemic as of February 2020. The household crowding index was calculated by dividing the number of persons living in the house by the number of rooms, excluding bathrooms and the kitchen [16]. The monthly income was divided into four levels: no income, low < 1000 USD, intermediate 1000–2000 USD, and high income > 2000 USD. Fear of poverty was also assessed and rated on a Likert scale from 0 (no fear) to 10 (extreme fear). The physical activity consisted of any sports and fitness workout practiced during confinement to stay fit and healthy; it was assessed using dichotomous questions (Yes/No).

The second part consisted of the following scales:

#### The Women’s Autonomy Index (WAI)

This index was created using questions adapted from a previous study [17]. It consisted of three questions: (1) Do you have the capacity to open and operate your own bank account?; (2) Do you have the capacity to meet the financial needs of your family?; and (3) What are the places where you can go alone (unaccompanied)? Questions 1 and 2 had three possible answers rated from 0 to 2, while question 3 had four possible answers and a score from 0 to 3. The total score ranged between 0 and 7, where a higher score indicates higher woman independence.

#### The Composite Abuse Scale Revised—Short Form (CASR-SF)

This 15-item scale is a self-report tool covering three domains of abuse (i.e., physical, sexual, and psychological) and evaluating abuse exposure and frequency [18]. It was developed by Ford-Gilboe et al. among 6278 adult Canadian women and had an internal consistency of 0.942 [18]. Moreover, correlations were moderate between the CASR-SF and the measures of depression, post-traumatic stress disorder, and coercive control [18]. The total score is calculated by summing the 15 responses rated from 0 to 6, with a higher score indicating a higher intensity/occurrence of abuse. Three subscales scores were derived from the total score, reflecting physical (4 items), sexual (2 items), and psychological (6 items) abuse [18]. The scale was dichotomized into non-abused (answering 0 to all questions) and abused (answering at least 1 to any question). Pr. Marilyn Ford-Gilboe, the author of the questionnaire, authorized its use in this study.

#### Perceived stress scale short version (PSS-4)

Three versions of the Perceived Stress Scale are available: PSS-14 (14 items), PSS-10 (10 items), and PSS-4 (4 items). The original 14-item instrument was in English; it consisted of seven positive items and seven negative items rated on a 5-point Likert scale [19]. It has demonstrated good reliability, with Cronbach’s alpha ranging from 0.75 to 0.91 [20]. The PSS has been translated into several languages and evaluated in various cultures and countries [20]. Chaaya et al. validated the Arabic version of the PSS-10 items in Lebanon among 268 women and found a good validity with an adequate internal consistency of 0.74 [21]. Almadi et al. validated another Arabic version of the PSS-14 in Jordan among 126 participants; the Cronbach’s alpha was 0.80, and the test–retest reliability had an intra-correlation coefficient of 0.90 [22]. In the current study, the 4-item version was used as a brief measure was required to measure the stress perception as a potential confounder. Answers are rated on a scale from 0 (never) to 4 (very often). The total score is calculated by summing the four responses and ranges from 0 to 16, with higher scores indicating more perceived stress [19].

#### Lebanese Anxiety Scale (LAS-10)

This 10-item self-report scale is used to screen for anxiety [23]. It was developed and validated in Lebanon by Hallit et al. among 1332 Lebanese adults and showed a good internal consistency of 0.857 and good sensitivity and specificity (77.5% and 70.8%, respectively). The positive predictive value of the LAS-10 score was 26.9%, and the negative predictive value was 95.2% [23]. Seven of the items are graded on a 5-point Likert scale (0 = Not present to 4 = very severe), and the remaining three, on a 4-point Likert scale (1 = almost never to 4 = almost always) [23]. The total score is calculated by summing all the responses, with higher scores indicating higher anxiety [23].

#### Patient Health Questionnaire (PHQ-9)

The PHQ-9 consists of nine questions used to screen for the presence and severity of depression [24]. Its diagnostic validity has been established in two studies involving 3000 patients in eight primary care clinics and 3000 patients in seven obstetrics-gynecology clinics, respectively [25, 26]. It had a sensitivity of 88% and a specificity of 88% for detecting major depressive disorder, in addition to adequate reliability and validity and a high internal consistency [25, 26]. In Lebanon, Sawaya et al. translated and validated the PHQ-9 among 186 Lebanese adult psychiatric patients [27]; the scale showed a high internal consistency (Cronbach’s alpha = 0.88) and an adequate factor analysis [27]. Based on a cutoff of 10, it had a sensitivity of 77% and a specificity of 46% for detecting depressive symptoms [27]. All items are rated from 0 (not at all) to 3 (nearly every day). The PHQ-9 total score ranges from 0 to 27, with higher scores reflecting a more severe depression.

### Statistical analysis

Statistical Package for the Social Sciences (SPSS) software version 25 (SPSS Inc., Chicago, IL, USA) was used for data analysis. No missing values was detected in the database. Cronbach’s alpha values were recorded for the reliability analysis of all scales. In the descriptive analysis, counts and percentages were used for categorical variables and means and standard deviations for continuous measures. The Women’s Autonomy Index was normally distributed according to the histogram and the kurtosis and skewness values. Two linear regressions were performed, taking the Women’s Autonomy Index as the dependent variable, and included the variables that showed a *p* value less than 0.05 in the bivariate analysis to minimize confounding. Also, a multivariate General Linear Model was conducted, comparing mental health variables between autonomous and non-autonomous women, after adjusting for sociodemographic characteristics. A value of *p* < 0.05 was considered statistically significant.

## Results

### Sample description

Table 1 presents the details of the sociodemographic and other characteristics of the participants. Of the 369 total sample, 72% answered the Arabic questionnaire, while 28% preferred the English version. The percentage of women with university education was higher than that of their partners, while employment and income were much higher among men. The majority of participants were from Mount Lebanon and Beirut (76.4%); the others were from North (11.9%), South (6.5%), and Beqaa (5.1%). The mean duration of confinement was 71.0 ± 42.8 days, and the mean fear of poverty was 5.8.

**Table 1:** Sociodemographic and other characteristics of the studied sample (N = 369)

	Woman’s response	Partner characteristics reported by woman
	Frequency (%)	Frequency (%)
*Education level*		
Primary	3 (0.8%)	18 (4.9%)
Complementary	11 (3.0%)	34 (9.2%)
Secondary	32 (8.7%)	64 (17.3%)
University	323 (87.5%)	253 (68.6%)
*Religion*		
Christian	115 (31.2%)	115 (31.2%)
Muslim	155 (42.0%)	159 (43.1%)
Druze	81 (22.0%)	80 (21.7%)
Atheist	2 (0.5%)	2 (0.5%)
Refused to answer	16 (4.3%)	13 (3.5%)
*Working status*		
Employed	221 (59.9%)	334 (90.5%)
Unemployed	148 (40.1%)	35 (9.5%)
*Monthly income*		
No income	102 (27.6%)	19 (5.1%)
Low	94 (25.5%)	71 (19.2%)
Intermediate	112 (30.4%)	168 (45.5%)
High	61 (16.5%)	111 (30.2%)
*Smoking status*		
Non smoker	254 (68.8%)	170 (46.1%)
Smoker	115 (31.2%)	199 (53.9%)
*Alcohol consumption*		
Yes	40 (10.8%)	148 (40.1%)
No	329 (89.2%)	221 (59.9%)
*Physical activity*		
Yes	157 (42.5%)	128 (34.7%)
No	212 (57.5%)	241 (65.3%)

	Mean ± SD	Mean ± SD
Age in years	32.5 ± 6.4	37.6 ± 7.2
Duration of confinement (days)	71.0 ± 42.8	
Fear of poverty	5.8 ± 3.2	

### Description of the scales used

The mean WAI was 4.32 ± 1.55 with a Cronbach’s alpha value of 0.328. The factor analysis of the scale is presented in Additional file 3: Table S1. In the absence of cut-off values for WAI, the median was considered a cut-off point for higher and lower autonomy. The mean total CASR-SF was 1.97 ± 5.83 with a Cronbach’s alpha value of 0.902 for the full scale. Cronbach's alpha values for the subscales of CASR-SF were 0.791 for the psychological abuse subscale, 0.759 for the physical abuse subscale, and 0.740 for the sexual abuse subscale. The factor analysis of the scale is presented in Additional file 3: Table S2. The mean PSS-4 was 7.35 ± 2.62 with a Cronbach’s alpha value of 0.484, and the mean LAS-10 scale was 14.44 ± 7.38 with a Cronbach’s alpha value of 0.890. The mean PHQ-9 was 7.02 ± 5.44 with a Cronbach’s alpha value of 0.893.

**Fig. 1:**
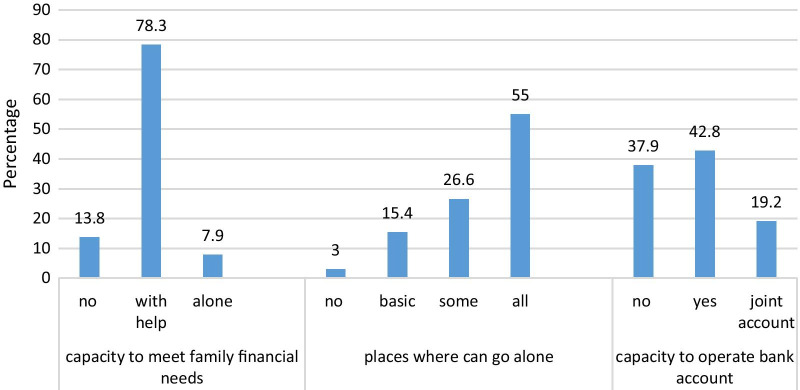
Women’s answers to the questions used to construct the Women’s Autonomy Index

Moreover, only 7.9% of women reported being able to meet the financial needs of their family, while 55.0% can go alone to any place, and 42.8% can operate a bank account without their partner (Fig. 1, Additional file 3: Table S3).

### Association of CASR-SF items with Women’s autonomy scale items

The results of the CASR-SF showed 15.2% of abuse, divided into sexual (1.8%), physical (1.8%), verbal (73.2%), physical/verbal (19.6%), and physical/verbal/sexual (3.6%).

Table 2 shows the association of all WAI aspects with the CASR-SF scale, dichotomized into abused (N = 96; 26%) versus not abused women (N = 273; 74%).

**Table 2:** Association of CASR-SF items with Women’s autonomy scale items

	Item 1: Capacity to meet family financial needs				
	No	With help	Alone	*p* value	
*CASR-SF total*					
No abuse	36 (70.6%)	215 (74.4%)	22 (75.9%)	0.825	
Abuse	15 (29.4%)	74 (25.6%)	7 (24.1%)		
*CASR-SF psychological*					
No abuse	37 (72.5%)	221 (76.5%)	22 (75.9%)	0.833	
Abuse	14 (27.6%)	68 (23.5%)	7 (24.1%)		
*CASR-SF physical*					
No abuse	47 (92.3%)	263 (91%)	28 (96.6%)	0.663	
Abuse	4 (7.8%)	26 (9%)	1 (3.4%)		
*CASR-SF sexual*					
No abuse	47 (92.2%)	275 (95.2%)	29 (100%)	0.301	
Abuse	4 (7.8%)	14 (4.8%)	0 (0%)		

	Item 2: Capacity to operate a bank account				
	No	Joint account	Yes	*p* value	
*CASR-SF total*					
No abuse	106 (75.7%)	62 (87.3%)	105 (66.5%)	**0.003**	
Abuse	34 (24.3%)	9 (12.7%)	53 (33.5%)		
*CASR-SF psychological*					
No abuse	109 (77.9%)	62 (87.3%)	109 (69%)	**0.009**	
Abuse	31 (22.1%)	9 (12.7%)	149 (31%)		
*CASR-SF physical*					
No abuse	129 (92.1%)	69 (97.2%)	140 (88.6%)	0.092	
Abuse	11 (7.9%)	2 (2.8%)	18 (11.4%)		
*CASR-SF sexual*					
No abuse	129 (92.1%)	69 (97.2%)	153 (96.8%)	0.135	
Abuse	11 (7.9%)	2 (2.8%)	5 (3.2%)		

	Item 3: Places where she can go alone				
	No	Basic	Some	All	*p* value
*CASR-SF total*					
No abuse	10 (90.9%)	43 (75.4%)	73 (74.5%)	147 (72.4%)	0.596
Abuse	1 (9.1%)	14 (24.6%)	25 (25.5%)	56 (27.6%)	
*CASR-SF psychological*					
No abuse	10 (90.9%)	43 (75.4%)	74 (75.5%)	153 (75.4%)	0.723
Abuse	1 (9.1%)	14 (24.6%)	24 (24.5%)	50 (24.6%)	
*CASR-SF physical*					
No abuse	11 (100%)	50 (87.7%)	92 (93.9%)	185 (91.1%)	0.400
Abuse	0 (0%)	7 (12.3%)	6 (6.1%)	18 (8.9%)	
*CASR-SF sexual*					
No abuse	11 (100%)	52 (91.2%)	97 (99%)	191 (94.1%)	0.102
Abuse	0 (0%)	5 (8.8%)	1 (1%)	12 (5.9%)	

Note: Values marked in Bold are significant

Regarding the CASR-SF total score, the results showed that among women who do not operate a bank account, 24.3% were abused, while among those who have a joint account, 12.7% experienced abuse, and among women who operate their own account, 33.5% were abused.

As for the CASR-SF psychological sub-scale, it was found that among women who do not operate a bank account, 22.1% endured abuse, while among those who have a joint account, 12.7% were abused, and among those who operate their own account 31% were abused (Table 2).

### Bivariate analysis: correlates of the WAI

Table 3 presents the bivariate analysis taking the WAI as a dependent variable. The results showed a significant association between high WAI scores and women and partners with high education levels and high monthly incomes. Additionally, lower WAI scores were significantly associated with higher stress, higher anxiety, higher sexual violence, and a higher total violence score.

**Table 3:** Bivariate analysis taking the Women’s Autonomy Index as the dependent variable

	Women’s Autonomy Index			
	Woman’s characteristics		Partner’s characteristics	
	r	*p* value	r	*p* value
Age	0.248	**< 0.001**	0.156	**0.003**
Age at marriage	0.290	**< 0.001**	0.019	0.721
Age at birth of first child	0.272	**< 0.001**	0.022	0.700
Duration between the first meeting and marriage	0.182	**< 0.001**		
Perceived stress (PSS-4)	− 0.117	**0.024**		
Anxiety scale (LAS-10)	− 0.204	**< 0.001**		
Depression scale (PHQ-9)	− 0.099	0.058		
CASR-SF total	− 0.106	**0.041**		
CASR-SF psychological	− 0.097	0.062		
CASR-SF physical	− 0.031	0.551		
CASR-SF sexual	− 0.131	**0.012**		

	Mean (SD)	*p* value	Mean (SD)	*p* value
*Education level*				
School education	2.65 (1.36)	**< 0.001**	3.85 (1.67)	**< 0.001**
University education	4.56 (1.43)		4.54 (1.45)	
Religion*				
Christian	4.80 (1.29)	**0.001**	4.80 (1.29)	**< 0.001**
Muslim	4.11 (1.69)		4.10 (1.68)	
Druze	4.01 (1.54)		4.00 (1.54)	
Other	4.55 (1.38)		4.73 (1.33)	
*Work status*				
Unemployed	3.71 (1.60)	** < 0.001**	4.17 (1.74)	0.540
Employed	4.73 (1.38)		4.34 (1.53)	
Monthly income*				
No income	3.33 (1.41)	** < 0.001**	3.79 (1.93)	**< 0.001**
Low income	4.08 (1.40)		3.75 (1.69)	
Intermediate income	4.98 (1.24)		4.23 (1.46)	
High income	5.15 (1.54)		4.94 (1.34)	
*Alcohol consumption*				
Yes	5.62 (0.80)	** < 0.001**	4.81 (1.30)	**< 0.001**
No	4.16 (1.55)		4.00 (1.62)	
Type of smoking*				
Cigarette	4.72 (1.37)	**0.025**	4.20 (1.49)	0.194
Waterpipe	4.06 (1.62)		4.44 (1.44)	
Cigarette and waterpipe	5.43 (1.27)		4.30 (2.00)	
Other	4.28 (1.59)		5.18 (0.98)	
*Physical activity*				
No	4.19 (1.62)	0.059	4.35 (1.56)	0.642
Yes	4.50 (1.45)		4.27 (1.54)	
Living region*				
Mount Lebanon	4.38 (1.50)	**0.023**		
Beirut	4.64 (1.58)			
North	4.32 (1.46)			
South	3.38 (1.74)			
Beqaa	4.16 (1.80)			
*Living place*				
Urban	4.45 (1.54)	**0.027**		
Rural	4.06 (1.57)			

Note: Values marked in Bold are significant

*Bonferroni Post-hoc analysis: religion of women: Christian versus Muslim *p* = 0.002; Christian versus Druze *p* = 0.003, Christian versus other *p* = 1.000, Muslim versus Druze *p* = 1.000, Muslim versus other *p* = 1.000, Druze versus other *p* = 1.000

Religion of partners: Christian versus Muslim *p* = 0.001; Christian versus Druze *p* = 0.002, Christian versus other *p* = 1.000, Muslim versus Druze *p* = 1.000, Muslim versus other *p* = 0.750, Druze versus other *p* = 0.528

Woman’s monthly income: no income versus low-income *p* = 0.001, no versus intermediate income *p* < 0.001, no versus high-income *p* < 0.001, low versus intermediate income *p* < 0.001, low versus high-income *p* < 0.001, intermediate versus high-income *p* = 1.000

Partner’s monthly income: no income versus low-income *p* = 1.000, no versus intermediate income *p* = 1.000, no versus high-income *p* = 0.013, low versus intermediate income *p* = 0.146, low versus high-income *p* < 0.001, intermediate versus high-income *p* = 0.001

Woman’s type of smoking: cigarette versus waterpipe *p* = 0.190, cigarette versus waterpipe/cigarette *p* = 0.869, waterpipe versus waterpipe/cigarette *p* = 0.081

Living region: Mount Lebanon versus Beirut *p* = 1.000, Mount Lebanon versus North *p* = 1.000, Mount Lebanon versus South *p* = 0.026, Mount Lebanon versus Beqaa *p* = 0.026, Beirut versus North *p* = 1.000, Beirut versus South *p* = 0.012, Beirut versus Beqaa *p* = 1.000, North versus South *p* = 0.164, North versus Beqaa *p* = 1.000, South versus Beqaa *p* = 0.991

### Multivariable analysis

The first linear regression taking the Women’s Autonomy Index as the dependent variable in the context of the woman’s characteristics showed that university education level (beta = 1.263), alcohol consumption (beta = 0.586), intermediate income level (beta = 0.702), high income (beta = 0.911), employment (beta = 0.559), and older age (beta = 0.033), were significantly associated with greater autonomy. Whereas, living in South Lebanon (beta = − 0.668) and being Druze (beta = − 323) were associated with lower woman autonomy (Table 4, Model 1).

**Table 4:** Multivariable analysis

Factor	Unstandardized beta	Standardized beta	95% CI	*p* value
*Linear regression 1 taking the Women’s Autonomy Index as dependent variable in the context of the woman’s characteristics*				
University education versus school education^a^	1.263	0.268	0.847; 1.679	< 0.001
Alcohol consumption (Yes vs. No^a^)	0.586	0.117	0.119; 1.053	0.014
Woman intermediate income versus no income^a^	0.702	0.207	0.381; 1.024	< 0.001
Woman high income versus no income^a^	0.911	0.218	0.504; 1.318	< 0.001
Woman employed versus unemployed^a^	0.559	0.176	0.263; 0.855	< 0.001
Woman age (years)	0.033	0.133	0.011; 0.054	0.003
Living in South Lebanon versus Mount Lebanon^a^	− 0.668	− 0.106	− 1.205; − 0.132	0.015
Woman Druze versus Christian^a^	− 0.323	− 0.086	− 0.638; − 0.008	0.044
*Linear regression 2 taking the Women’s Autonomy Index as dependent variable in the context of the partner’s characteristics*				
High monthly income versus no income^b^	0.628	0.185	0.285; 0.972	< 0.001
Alcohol consumption (yes vs. no^b^)	0.661	0.208	0.347; 0.975	< 0.001
University education versus school education^b^	0.420	01.125	0.082; 0.758	0.015
Woman Druze versus Christian^b^	− 0.401	− 0.106	− 0.761; − 0.040	0.030
Age	0.021	0.098	0.001; 0.042	0.050

^a^Variables entered: region of living, work status, education level, religion, physical activity, income, age, alcohol consumption

^b^Variables entered: partner’s alcohol consumption, partner’s education level, partner’s religion, partner’s work status, partner’s income

Variables that were not entered in the model (*p* values not significant): partner’s work status, partner’s type of smoking, partner’s physical activity, partner’s age at marriage, partner’s age at birth of first child

The second linear regression taking the Women’s Autonomy Index as the dependent variable in the context of the partner’s characteristics showed that a high income (beta = 0.628), university education level (beta = 0.420), alcohol consumption (beta = 0.661), and older age (beta = 0.021) were significantly associated with higher woman autonomy while being Druze (beta = − 0.401) was significantly associated with lower woman autonomy (Table 4, Model 2).

**Fig. 2:**
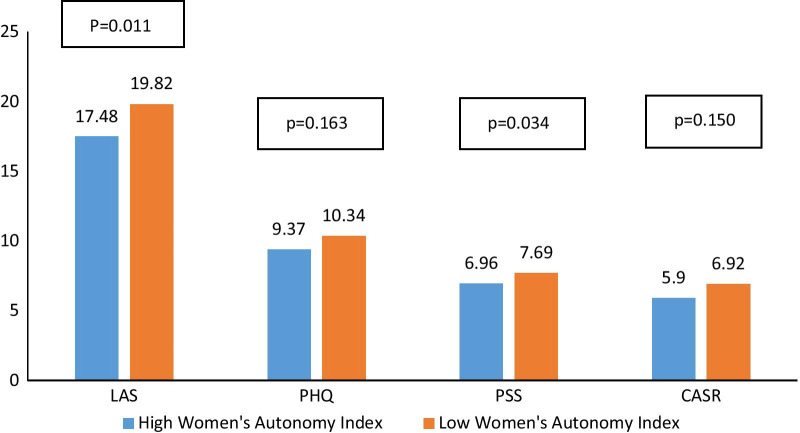
Adjusted means of psychological variables according to the Women’s Autonomy Index. *LAS* Lebanese Anxiety Scale, *PHQ* Patient Health Questionnaire for depression measurement, *CASR* Composite Abuse Scale Revised, *PSS-4* Perceived Stress Scale

Figure 2 displays the association of psychological variables with WAI scores, adjusted for woman’s age, woman’s age at marriage, duration between first meeting and marriage, the region of living, work status, education level, religion, physical activity, income, alcohol consumption, partner’s alcohol consumption, partner’s religion, partner’s work status, partner’s income. Women with low autonomy had significantly higher LAS-10 and PSS-4 scores (Additional file 3: Table S4).

## Discussion

To our knowledge, this study is the first in Lebanon related to women’s autonomy, its associated factors, and mental health aspects. The vast majority (more than 90%) of participants were not able to fulfill the economic needs of their family, while around half of them could not go to places they want, or operate a bank account without their partner, although our sample included highly educated women with easy access to the Internet (online survey). Nevertheless, compared to non-abused, abused women were more likely to have their own bank account, probably as a coping mechanism. A possible explanation of this result could be that most local and international organizations advocating against domestic violence encourage abused women to protect themselves financially, when possible, through several means, including opening and running a personal bank account, which is relatively easy in Lebanon, where the authorization of a tutor is not required [28, 29].

It is also noteworthy that the percentage of women with university education was higher than that of their partners, while employment and income were much higher among men. These results are in agreement with the Lebanese social-economic context [30]: despite women being more educated, the majority still work at the lower levels of the economic pyramid, even though 30–50% of the workforce consists of females, and the economic empowerment of woman plays a significant role in facilitating the achievement of a higher level of economic welfare [15, 31]. Inspired by religious discourse, this legal structure classifies Lebanese women as second-class citizens, treating them as minors in decisions related to governing their own lives [32].

In this complex structure, our study also showed that personal and partner-related factors were associated with the autonomy of women, from the financial, operational, and social facets (the three questions of the index). Women’s autonomy was positively associated with a university education, alcohol consumption, intermediate to high-income level vs. lower-income categories), employment, and age. Oppositely, some cultures seem to be associated with a lower autonomy of women, such as living in South Lebanon and being Druze. Our results are consistent with those of previous studies showing that, among Lebanese women, ambitions, values, and priorities related to education and economic independence differ according to religious and cultural differences [8, 31]. The patriarchal character of the Lebanese society might be the primary reason for these differences [33]. As for age and education, our results are similar to those found in other developing countries, such as Ethiopia [1], India [6], Nepal [7], and Uganda [34].

Regarding the characteristics of partners, high income, university education, alcohol consumption, and older age are associated with the autonomy of women, while being Druze was inversely associated with it. Our results revealed that the characteristics of partners are as important as those of women. Thus, education, income, and cultural background of the partner are influential drivers of autonomy; our findings are consistent with those of studies in other developing countries, such as Uganda [34], Nepal [10], and Ethiopia [11].

Women’s autonomy was significantly associated with lower perceived stress and lower anxiety, in agreement with previous findings. The evidence from a systematic review suggests that lower control or autonomy of women (e.g., lack of freedom of movement outside the home) was associated with poorer mental health for women after adjusting for their socioeconomic circumstances [35]. High agency in household decision-making has direct implications for women’s control over household material resources, such as income and assets, which may give women additional resources to cope with adverse conditions and life stressors [36]. Some autonomy components may be associated with experiencing anxiety in different situations (e.g., related to others’ distress or presenting one’s personal views) [37]; also, low autonomy in decision-making is related to higher levels of common mental disorders, including anxiety and depression [38]. In Egypt, anxiety was related to premarital variables in particular, such as education and socioeconomic level, which in turn are related to the autonomy of women [39].

The association of autonomy with depression and abuse yielded positive but non-significant results. Social and economic empowerment were similarly correlated to depressive symptoms in Afghanistan [40]. A study comparing Turkish to German women found that autonomy was associated with higher levels of mental health in both Turkish and German women (lower depression) [41]. Thus, empowerment may protect women against intimate partner violence overall, but this relationship can be reversed at the micro-level [42].

### Study limitations

This study has several limitations. Its cross-sectional design cannot show causality but only generates hypotheses. A selection bias is suspected, given that only educated women with higher socioeconomic status and internet access could answer the online survey, which leads to the underestimation of our results. Indeed, data show that 57% of Lebanese women have university education compared to 87.5% in our sample [30]. Further underestimation may be linked to non-differential information bias due to self-declared answers with possibilities of recall or social desirability biases. Finally, although a multivariate analysis was conducted, the possibility of residual confounding cannot be ruled out. Further prospective studies that take into account these pitfalls are necessary to confirm our results.

## Conclusion

This study showed that, in Lebanon, the autonomy and empowerment of women depend on several personal and partner-related characteristics (education, socioeconomic status, age), in addition to the cultural (geographic and religious) environment. Furthermore, low autonomy is associated with higher perceived stress and anxiety and probable depression and domestic abuse.

## Supplementary Information

**Additional file 1.** STROBE Statement checklist.

**Additional file 2.** Woman Autonomy Index questionnaire.

**Additional file 3.** **Table 1:** Factor structured of the Women autonomy index. **Table 2:** Factor structured of the Composite Abuse Scale (Revised)—Short Form (CASR-SF). **Table 3:** Description of the women autonomy index items. **Table 4:** Association between the women autonomy index and psychological factors.

## Data Availability

Data can be made available under reasonable request form the corresponding author.

## Abbreviations

WAI: Women’s Autonomy Index
USD: United States dollar
CASR-SF: The Composite Abuse Scale Revised—Short Form
PSS-4: Perceived stress scale short version
LAS-10: Lebanese Anxiety Scale
PHQ-9: Patient Health Questionnaire
SPSS: Statistical Package for Social Sciences
CI: Confidence interval
