# Supplementary material for: Factors related to autonomy among Lebanese women: a web-based cross-sectional study
Source: BMC Womens Health. 2021 Oct 20;21:369. doi: 10.1186/s12905-021-01501-3 (PMC8527961; doi:10.1186/s12905-021-01501-3)
Supplement: Supplementary file 2 — Additional file 2. Woman Autonomy Index questionnaire. [file 12905_2021_1501_MOESM2_ESM.docx]

**WOMAN AUTONOMY INDEX QUESTIONNAIRE**

**Factors Related to Autonomy Among Lebanese Women:
A Web-Based Cross-Sectional Study**

**Sandrella Bou Malhab^1^ , Hala Sacre^1^, Diana Malaeb^1,2^, Nathalie Lahoud^1,3,4^, Dalia Khachman^5^, Joelle Azzi^3^, Chadia Haddad^1,6,7*^, Pascale Salameh^1,4,8*^**

1. INSPECT-LB (Institut National de Santé Publique, d’Épidémiologie Clinique et de Toxicologie-Liban), Beirut, Lebanon
2. School of Pharmacy, Lebanese International University, Beirut, Lebanon
3. Faculty of Public Health, Lebanese University, Fanar, Lebanon
4. Faculty of Pharmacy, Lebanese University, Beirut, Lebanon
5. Clinical and Epidemiological Research Laboratory, Faculty of Pharmacy, Lebanese University, Hadat, Lebanon
6. Research Department, Psychiatric Hospital of the Cross, JalEddib, Lebanon
7. INSERM, Univ. Limoges, CH Esquirol, IRD, U1094 Tropical Neuroepidemiology, Institute of Epidemiology and Tropical Neurology, GEIST, Limoges, France
8. University of Nicosia Medical School, Nicosia, Cyprus

***Last Co-authors**

**Corresponding authors:** Chadia Haddad. Psychiatric Hospital of the Cross, P.O. Box 60096, Jall-Eddib, Lebanon. Email address: [Chadia_9@hotmail.com](mailto:Chadia_9@hotmail.com)

**Section 1: Sociodemographic and lifestyle characteristics**

| **Woman** | | | |  | **Partner** | | |
| --- | --- | --- | --- | --- | --- | --- | --- |
| 1- | |  | Age: ___________ years | | 9- Age----------------------years | | |
|  | 2- Has your doctor ever told you that you | | | | 10- | Educational level | |
|  |  | have infertility problems | | |  | Illiterate | |
|  |  |  |  | No |  | Primary | |
|  |  |  |  | I don’t know |  | Complementary | |
|  |  |  |  | Yes |  | Secondary | |
|  |  |  |  |  |  | University | |
| 3- | |  | Educational level | | 11- | Religion | |
|  |  |  |  |  |  | Christian | |
|  |  | Illiterate | | |  | Muslim | |
|  |  | Primary | | |  | Druze | |
|  |  | Complementary | | |  | Atheist | |
|  |  | Secondary | | |  | Other______ | |
|  |  | University | | |  | Refuse to answer | |
| 4- | |  | Living place | | 12- | Working status during the quarantine | |
|  |  | Urban | | |  | Working from home | |
|  |  | Rural | | |  | Going to work | |
| 5- | |  | Region | |  | Unemployed | |
|  |  |  |  |  |  | Never works | |
|  |  | Mount Lebanon | | |  |  |  |
|  |  | Beirut | | | 13- | Monthly income (1$=1.500 LL) | |
|  |  | North | | |  | No income | |
|  |  | South | | | Low (<1,500,000 LL) | | |
|  |  | Bekaa | | | Intermediate (1,500,000 – 3,000,000 LL) | | |
| 6- | |  | Religion | | High (>3,000,000 LL) | | |
|  |  |  |  |  |  |  |  |

Christian Muslim Druze

Atheist

Other______

Refuse to answer

7- Working status during the quarantine

|  | Working from home | |  |  |  |
| --- | --- | --- | --- | --- | --- |
|  | Going to work | |  |  |  |
|  | Unemployed | |  |  |  |
|  | Never works | |  |  |  |
| 8- |  | Monthly income (1$=1.500 LL) |  |  |  |
| No income | | |  |  |  |
| Low (<1,500,000 LL) | | |  |  |  |
| Intermediate (1,500,000 – 3,000,000 LL) | | |  |  |  |
| High (>3,000,000 LL) | | |  |  |  |
|  |  | |  |  | |
| 14- | Smoking status: | | 16- | Smoking status: | |
|  |  | Regular smoker |  |  | Regular smoker |
|  |  | Occasional smoker |  |  | Occasional smoker |
|  |  | Previous smoker |  |  | Previous smoker |
|  |  | Non-smoker |  |  | Non-smoker |
| 15- If you are a current smoker, what type of | | | 17- | If your partner are a current smoker, what type of | |
|  | tobacco are you smoking? | |  | tobacco is he smoking? | |
|  |  | Cigarette |  |  | Cigarette |
|  |  | Waterpipe |  |  | Waterpipe |
|  |  | Ciagrette and waterpipe |  |  | Ciagrette and waterpipe |
|  |  | Other______________________ |  |  | Other__________________________ |
|  |  | |  |  | |
| 18- | Alcohol consumption: | | 19- | Alcohol consumption: | |
|  |  | Regular |  |  | Regular |
|  |  | Occasional |  |  | Occasional |
|  |  | Previous alcohol drinking |  |  | Previous alcohol drinking |
|  |  | Never |  |  | Never |
| 20- Substance use (cocaine, heroin, | | | 21- Substance use (cocaine, heroin, marijuana, | | |
|  | marijuana, cannabis…) | |  | cannabis…) | |
|  |  | Yes |  | Yes |  |
|  |  | No |  | No |  |
| 22- Do you practice any kind of physical | | | 23- Does he practice any kind of physical activity | | |
|  | activity during the confinement: | |  | during the confinement: | |
|  |  | Yes |  | Yes |  |
|  |  | No |  | No |  |
| 24- Number of persons living in the house | | |  |  |  |
|  | including you: -------------- | |  |  |  |
| 25- Number of rooms besides kitchen and | | |  |  |  |
|  | bathrooms: ------------------- | |  |  |  |

27- Duration of marriage: _________years

28- Your age at marriage: _________years

29- Your partner’s age at marriage: _________years

30- Duration between first meeting and marriage: _________years

31- Number of times you got pregnant during lifetime ______________

32- When getting pregnant, was any of your pregnancies induced by:

Medical intervention such as in-vitro fertilization (IVF) Artificial treatment

No, all were natural

33- Do you have children: Yes/No

34- If yes, number of alive children ______________

35- If yes, your age at the birth of your first child____________

36- If yes, your partner’s age at the birth of your first child ____________

37- Any history of pregnancy negative outcome (ever had a neonatal death, miscarriage, and or stillbirth)

Yes No

38- Any history of pregnancy termination

Yes No

39- History of past unintended pregnancy

Yes No

40- Have you ever faced any pregnancy related complications (such as severe bleeding, unsafe abortion)?

Yes No

41- Number of induced abortions if ever : -----------

42- Number of natural abortions if ever:____________

**Section 2: Scales used**

**Women Autonomy Index**

44- Capacity of woman to operate bank account

Does not own or operate Owns and operate

Operates a joint account with her partner

45- Capacity of woman to meet financial needs for her family

No capacity

Capacity, with help Capacity, alone

46- Places that woman can go alone

No place

Basic places (Work/Supermarket/essential needs) Some places (in addition to basic places)

All places

**Perceived stress scale 4 (PSS-4)**

|  | Never  (0) | Almost  never  (1) | Sometimes  (2) | Fairly  often  (3) | Very often  (4) |
| --- | --- | --- | --- | --- | --- |
| 1. In the last month, how often have   you felt that you were unable to control the important things in your life? |  |  |  |  |  |
|  |  |  |  |  |  |
|  |  |  |  |  |  |
| 1. In the last month, how often have   you felt confident about your ability to  handle your personal problems? |  |  |  |  |  |
|  |  |  |  |  |  |
|  |  |  |  |  |  |
| 1. In the last month, how often have   you felt that things were going your way? |  |  |  |  |  |
|  |  |  |  |  |  |
|  |  |  |  |  |  |
| 1. In the last month, how often have   you felt difficulties were piling up so  high that you could not overcome them? |  |  |  |  |  |

| **Lebanese anxiety Scale (LAS)** | | | | | |
| --- | --- | --- | --- | --- | --- |
| **Items** | **Not present** | **Mild** | **Moderate** | **Severe** | **Very severe** |
| 1. Insomnia   (Difficulty in falling asleep, broken sleep, unsatisfying sleep and fatigue on waking, dreams, nightmares, night terrors) | 0 | 1 | 2 | 3 | 4 |
| 1. Tension   (Feelings of tension, fatigability, startle response, moved to tears easily, trembling, feelings of restlessness, inability to relax.) | 0 | 1 | 2 | 3 | 4 |
| 1. Somatic (muscular)   (Pains and aches, twitching, stiffness, myoclonic jerks, grinding of teeth, unsteady voice, increased muscular tone) | 0 | 1 | 2 | 3 | 4 |
| 1. Anxious mood   (Worries, anticipation of the worst, fearful anticipation, irritability) | 0 | 1 | 2 | 3 | 4 |
| 1. Depressed mood   (Loss of interest, lack of pleasure in hobbies, depression, early waking, diurnal swing). | 0 | 1 | 2 | 3 | 4 |
| 1. Fears   (Of dark, of strangers, of being left alone, of animals, of traffic, of crowds) | 0 | 1 | 2 | 3 | 4 |
| 1. Intellectual   (Difficulty in concentration, poor memory) | 0 | 1 | 2 | 3 | 4 |
| 1. I feel inadequate | Almost never (1) | Sometimes (2) | Often (3) | Almost always (4) |  |
| 1. I feel that difficulties are piling up so that I cannot overcome them | Almost never (1) | Sometimes (2) | Often (3) | Almost always (4) |  |
| 1. I feel indecisive | Not at all (1) | Somewhat (2) | Moderately so (3) | Very much so (4) |  |

| **PATIENT HEALTH QUESTIONNAIRE (PHQ-9)** | | | | |
| --- | --- | --- | --- | --- |
|  | Not at all | Several  days | More than  half the  days | Nearly  every day |
| 1. Little interest or pleasure in doing things |  |  |  |  |
| 1. Feeling down, depressed, or hopeless |  |  |  |  |
| 1. Trouble falling or staying asleep, or sleeping too much |  |  |  |  |
| 1. Feeling tired or having little energy |  |  |  |  |
| 1. Poor appetite or overeating |  |  |  |  |
| 1. Feeling bad about yourself or that you are a failure or have let yourself or your family down |  |  |  |  |
| 1. Trouble concentrating on things, such as reading the newspaper or watching television |  |  |  |  |
| 1. Moving or speaking so slowly that other people could have noticed. Or the opposite being so figety or restless that you have been moving around a lot more than usual |  |  |  |  |
| 1. Thoughts that you would be better off dead, or of hurting yourself |  |  |  |  |

**COMPOSITE ABUSE SCALE REVISED - SHORT FORM (CASR-SF)**

These questions ask about your experiences in adult intimate relationships. By adult intimate relationship we mean a current or former husband, partner or boyfriend/girlfriend for longer than one month.

1- Have you ever been in an adult intimate relationship? (Since you were

16 years of age)

1. Yes
2. No – Skip out of remaining questions 2- Are you currently in a relationship?
   1. Yes
   2. No Go to Q4

3- Are you currently afraid of your partner?

1. Yes
2. No

4- Have you ever been afraid of any partner?

1. Yes

b.No

We would like to know if you experienced any of the actions listed below from any current or former partner or partners. If it ever happened to you, please tell us how often it usually happened in the past 12 months.

| My partner (s) | | Has this | |  |  |  |  |  |  |
| --- | --- | --- | --- | --- | --- | --- | --- | --- | --- |
|  |  | ever | | IF YES, how often did it happen in the past 12 months? | | | | | |
|  |  | happened | |  |  |  |  |  |  |
|  |  |  |  |  |  |  |  |  |  |
|  |  | to you? | |  |  |  |  |  |  |
|  |  | No | Yes | Not in | Once | A | Monthly | Weekly | Daily/almost |
|  |  |  |  | the past |  | few |  |  | daily |
|  |  |  |  | 12 |  | times |  |  |  |
|  |  |  |  | months |  |  |  |  |  |
| 5- | Blamed |  |  |  |  |  |  |  |  |
| me for causing | |  |  |  |  |  |  |  |  |
| their violent | |  |  |  |  |  |  |  |  |
| behavior | |  |  |  |  |  |  |  |  |
| 6- | Shook, |  |  |  |  |  |  |  |  |
| pushed, grabbed | |  |  |  |  |  |  |  |  |
| or threw me | |  |  |  |  |  |  |  |  |
| 7- | Tried to |  |  |  |  |  |  |  |  |
| convince my | |  |  |  |  |  |  |  |  |

family, children

or friends that I

am crazy or tried

to turn them

against me

8- Used or

threatened to use

a knife or gun or

other weapon

to harm me

9- Made me

perform sex acts

that I did not

want to perform

10- Followed

me or hung

around outside

my home or

work

11- Threatened

to harm or kill

me or someone

close to me

12- Choked

me

13- Forced or

tried to force me

to have sex

14- Harassed

me by phone,

text, email or

using social

media

15- Told me I

was crazy,

stupid or not

good enough

16- Hit me

with a fist or

object, kicked or

bit me

17- Kept me

from seeing or

talking to my

family or friends

18- Confined

or locked me in

a

room or other

space

19- Kept me

from having

access to a job,

money or

financial

resources
