# Supplementary material for: Factors related to autonomy among Lebanese women: a web-based cross-sectional study
Source: BMC Womens Health. 2021 Oct 20;21:369. doi: 10.1186/s12905-021-01501-3 (PMC8527961; doi:10.1186/s12905-021-01501-3)
Supplement: Supplementary file 3 — Additional file 3. Table 1: Factor structured of the Women autonomy index. Table 2: Factor structured of the Composite Abuse Scale (Revised)—Short Form (CASR-SF). Table 3: Description of the women autonomy index items. Table 4: Association between the women autonomy index and psychological factors. [file 12905_2021_1501_MOESM3_ESM.docx]

**Supplementary Files**

(Questionnaire)

**Supplementary Tables**

| **Supplementary table 1: Factor structured of the Women autonomy index** | |
| --- | --- |
|  | **Factor 1** |
| Capacity to meet family financial needs | 0.764 |
| Places where she can go alone | 0.553 |
| Capacity to operate a bank account | 0.678 |
| **Variance explained (%)** | 44.91% |

| **Supplementary table 2: Factor structured of the Composite Abuse Scale (Revised)—Short Form (CASR-SF)** | | | |
| --- | --- | --- | --- |
|  | **Factor 1** | **Factor 2** | **Factor 3** |
| Choked me | 0.951 |  |  |
| Confined or locked me in a room or other space | 0.891 |  |  |
| Forced or tried to force me to have sex | 0.748 |  |  |
| Kept me from having access to a job, money or financial resources | 0.656 |  |  |
| Kept me from seeing or talking to my family or friends | 0.623 |  |  |
| Hit me with a fist or object, kicked or bit me | 0.549 |  |  |
| Tried to convince my family, children or friends that I am crazy or tried to turn them against me |  | 0.863 |  |
| Blamed me for causing their violent behavior |  | 0.819 |  |
| Told me I was crazy, stupid or not good enough |  | 0.670 |  |
| Shook, pushed, grabbed or threw me |  | 0.473 |  |
| Used or threatened to use a knife or gun or other weapon to harm me |  |  | 0.890 |
| Threatened to harm or kill me or someone close to me |  |  | 0.802 |
| Followed me or hung around outside my home or work |  |  | 0.653 |
| Harassed me by phone, text, email or using social media |  |  | 0.556 |
| Made me perform sex acts that I did not want to perform |  |  | 0.408 |
| **Variance explained (%)** | 50.65 | 8.85 | 8.26 |

| **Supplementary Table 3: Description of the women autonomy index items** | |
| --- | --- |
|  | **Frequency (%)** |
| **Capacity to meet financial needs** |  |
| No | 51 (13.8%) |
| With help | 289 (78.3%) |
| Alone | 29 (7.9%) |
| **Places where can go alone** |  |
| No | 11 (3.0%) |
| Basic | 57 (15.4%) |
| Some | 98 (26.6%) |
| All | 203 (55.0%) |
| **Capacity to operate bank account** |  |
| No | 140 (37.9%) |
| Yes | 158 (42.8%) |
| Joint Account | 71 (19.2%) |

| **Supplementary Table 4: Association between the women autonomy index and psychological factors** | | |
| --- | --- | --- |
|  | **High WAI** | **Low WAI** |
|  | **Mean ± SE** | **Mean ± SE** |
| **Anxiety (LAS-10)** | 17.48 ± 2.92 | 19.82 ± 2.99 |
| *p-value* | 0.011 | |
| **Depression (PHQ-9)** | 9.37 ± 2.21 | 10.34 ± 2.26 |
| *p-value* | 0.163 | |
| **Stress (PSS-4)** | 6.96 ± 1.09 | 7.69 ± 1.12 |
| *p-value* | 0.034 | |
| **Composite abuse scale (CASR-SF)** | 5.90 ± 2.26 | 6.92 ± 2.31 |
| *p-value* | 0.150 | |
| WAI: women autonomy index, SE: standard error | | |
